# Cell-Derived Extracellular Matrix for Tissue Engineering and Regenerative Medicine

**DOI:** 10.3389/fbioe.2020.602009

**Published:** 2020-12-03

**Authors:** Marisa Assunção, Dorsa Dehghan-Baniani, Chi Him Kendrick Yiu, Thomas Später, Sebastian Beyer, Anna Blocki

**Affiliations:** ^1^School of Biomedical Sciences, Faculty of Medicine, The Chinese University of Hong Kong, Shatin, Hong Kong; ^2^Institute for Tissue Engineering and Regenerative Medicine, The Chinese University of Hong Kong, Shatin, Hong Kong; ^3^Institute for Clinical and Experimental Surgery, University of Saarland, Saarbrücken, Germany; ^4^Department of Biomedical Engineering, Faculty of Engineering, The Chinese University of Hong Kong, Shatin, Hong Kong; ^5^Department of Orthopaedics and Traumatology, Faculty of Medicine, The Chinese University of Hong Kong, Shatin, Hong Kong

**Keywords:** cell-derived extracellular matrix, stem cell niche, cell differentiation, tissue engineering, regenerative medicine, skeletal repair, cardiovascular repair, cell-extracellular matrix interactions

## Abstract

Cell-derived extracellular matrices (CD-ECMs) captured increasing attention since the first studies in the 1980s. The biological resemblance of CD-ECMs to their *in vivo* counterparts and natural complexity provide them with a prevailing bioactivity. CD-ECMs offer the opportunity to produce microenvironments with costumizable biological and biophysical properties in a controlled setting. As a result, CD-ECMs can improve cellular functions such as stemness or be employed as a platform to study cellular niches in health and disease. Either on their own or integrated with other materials, CD-ECMs can also be utilized as biomaterials to engineer tissues *de novo* or facilitate endogenous healing and regeneration. This review provides a brief overview over the methodologies used to facilitate CD-ECM deposition and manufacturing. It explores the versatile uses of CD-ECM in fundamental research and therapeutic approaches, while highlighting innovative strategies. Furthermore, current challenges are identified and it is accentuated that advancements in methodologies, as well as innovative interdisciplinary approaches are needed to take CD-ECM-based research to the next level.

## Introduction

The extracellular matrix (ECM) is the non-cellular component present in all connective tissues and has a composition specific for each tissue. It is comprised of a complex and highly organized three-dimensional macromolecular network of biomolecules. These include fibrous proteins (such as collagens) and glycosaminoglycan (GAG)-based components. Fibrous ECM components form the backbone of the polymer network, thereby providing shape/stability and tensile strength to tissues. They also regulate cell adhesion and support cell migration. GAG-based components fill the interstitial space, ensuring hydration and lubrication of tissues, and acting as a reservoir and modulator of cytokine signaling (Theocharis et al., 2016; Yong et al., 2020).

ECM-driven communication arises from a complex combination of biochemical, topological and biomechanical cues, facilitating a reciprocal dialogue with cells, which can respond via remodeling of the ECM. This multi-dimensional signaling enables the ECM to guide intricate cellular and tissue processes such as homeostasis, healing and regeneration (Kaukonen et al., 2017).

## ECM as a Biomaterial

The ECM is a biomaterial designed by nature that underwent over 600 million years of material optimization (Ozbek et al., 2010). It serves as a blueprint for many man-made biomimetic biomaterials. Nonetheless, these materials represent oversimplified versions of the ECM that are not able to replicate its complex bioactivity (Kaukonen et al., 2017). As a result, ECM derived from decellularized tissues, remains one of the most successful biomaterials in clinics (Hussey et al., 2018).

Unfortunately, tissue-derived ECM faces various challenges to its clinical application. The limited availability of human cadaveric tissue leads to the use of animal tissue-derived ECM as an alternative source. Especially the incomplete decellularization of tissue carries the risk of disease transmission and immunological rejection. Some ECMs are plainly not available, since some specific tissues are hard to isolate (e.g., stem cell niches). Further, tissue-derived ECM is set in its composition, therefore cannot be customized in its bioactivity toward a specific application (Aamodt and Grainger, 2016).

As cell-derived ECM (CD-ECM) partially recapitulates the complex biological machinery of native tissue (Ahlfors and Billiar, 2007), it can address many of the tissue-derived ECM’s limitations. It can be derived from human cell cultures by gentle decellularization to remove immunogenic components, while preserving its bioactivity. ECM-synthesizing cells can be standardized and pre-screened (Sharma et al., 2020), minimizing the risk of disease transmission. Deriving ECM *in vitro* provides the opportunity to select appropriate ECM-producing cell types, further modify them (e.g., genetically) and expose them to specific stimuli, thus enabling the creation of ECM with desired properties (Maia et al., 2020). CD-ECM is therefore an incredibly versatile material to be used in physiological studies and therapeutic approaches.

## Methodologies to Generate CD-ECM

Stromal cell-derived ECMs are rich in collagens (Antebi et al., 2015), while endothelial/epithelial CD-ECMs contain a laminin-rich basement membrane-like ECM (Davis and Senger, 2005). CD-ECM can be generated by culturing cells scaffold-free in 2D and 3D cultures (Serebriiskii et al., 2008; Hoshiba et al., 2016; Sharma et al., 2020). Alternatively, cells can also be seeded within hydrogels or scaffolds, forming hybrid CD-ECM-based materials (Sart et al., 2014; Suhaeri et al., 2018).

### Facilitating ECM Deposition *in vitro*

Slow ECM assembling kinetics *in vitro* necessitate long cell culture periods up to several weeks to harvest sufficient CD-ECM amounts for the desired application (Bourget et al., 2012). This can be improved by adjusting culture conditions (Hoshiba, 2017).

The most essential supplement for robust ECM deposition is ascorbate, a cofactor of lysyl hydroxylase and prolyl hydroxylase, essential enzymes in collagen fibrillogenesis (Pinnell, 1985). Collagen type I is the most prominent ECM component and its deposition increases the overall yield of CD-ECM and improves its mechanical properties. Nonetheless, rapid degradation of ascorbate (Grinnell et al., 1989) calls for frequent media changes, thereby discarding the not-yet deposited ECM components. A stable form of ascorbate (2-phospho-L-ascorbate) can reduce the frequency of medium replacements (Chen et al., 2011).

The yield of deposited ECM can be amplified by introducing macromolecules, which emulate the crowded conditions present *in vivo.* The biophysical principle of macromolecular crowding (MMC) relies on macromolecules occupying space, thereby increasing the effective concentration of other molecules and the thermodynamic activity of the system. This has profound effects on protein folding, molecular interactions and enzyme kinetics (Chen et al., 2011). In particular, under MMC more ECM can be deposited within 1 week than after several weeks under non-crowded conditions. Most commonly used “crowders” are Ficoll, carrageenan, polyvinylpyrrolidone and dextran sulfate (Lareu et al., 2007; Lu et al., 2011; Blocki et al., 2015; Gaspar et al., 2019), albeit dextran sulfate was recently found to act as a precipitating agent, independent of MMC (Assunção et al., 2020).

Culturing cells with low serum concentration (<1% v/v) was also beneficial, as serum carries exogenous matrix metalloproteases that degrade ECM and imbalance the ECM’s natural remodeling rate (Satyam et al., 2014; Kumar et al., 2015). Furthermore, hypoxia was shown to induce synthesis of ECM richer in collagenous proteins and angiogenic factors, as seen in fibroblasts (Distler et al., 2007; Kumar et al., 2018) and mesenchymal stem cells (MSCs) (Cigognini et al., 2016; Du et al., 2017).

### Decellularization and Processing of CD- ECM

**FIGURE 1:**
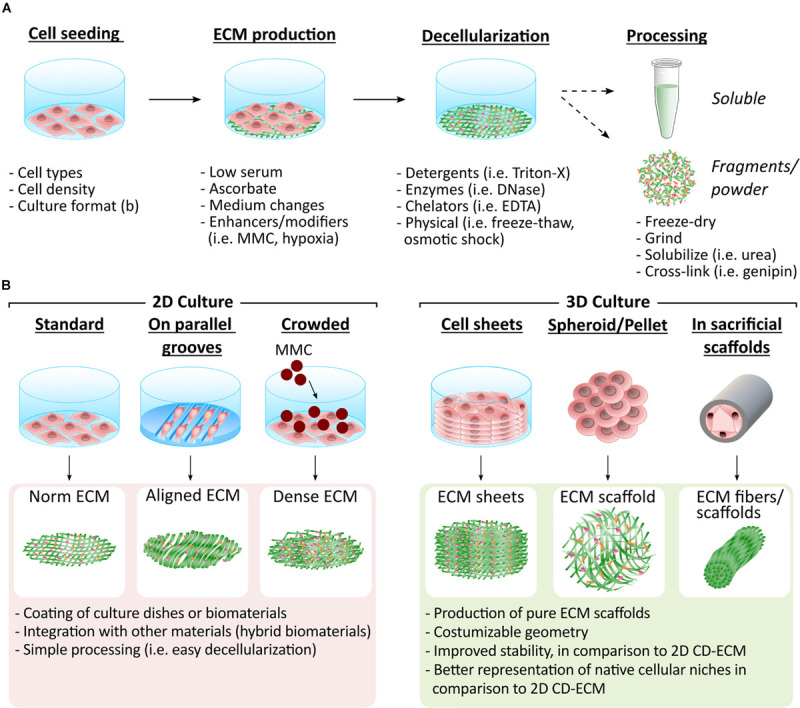
Methodologies to generate CD-ECM in different formats. **(A)** CD-ECMs are synthesized by different cell types (i.e., MSCs and fibroblasts). Culture conditions are adjusted to facilitate ECM deposition by e.g., introducing MMC or hypoxia into cell culture. The assembled ECM is then gently decellularized, while preserving the ECM’s integrity as much as possible. The resulting CD-ECM can be used in its original format or further processed. **(B)** The arrangement in which the ECM producing cells are cultured determines the format of the CD-ECM material. Which type of presentation is most advantageous depends on the desired application. Easiest decellularization can be achieved in 2D cultures. The resulting CD-ECM is suitable for coating of culture dishes and biomaterial surfaces or can be further processed and integrated with other biomaterials, such as hydrogels. Integration of CD-ECM with other materials provides the opportunity to combine the bioactivity of the ECM with desired geometries and mechanical properties. CD-ECM assembled in 3D recapitulates native cellular niches more closely. It can thus be utilized to engineer improved tissue models and ECM 3D scaffolds with desired geometries. Various techniques exist that enable the construction of 3D scaffolds based on CD-ECM.

CD-ECMs are usually generated in a small format, permitting gentle decellularization methods with focus on maintaining architecture and bioactivity. Most methods use detergents, enzymes, chelating agents, mechanical approaches and combinations thereof (Figure 1A; Woods and Gratzer, 2005; Faulk et al., 2014; Levorson et al., 2014; Gilpin and Yang, 2017). Complete decellularization is further achieved by removing genetic material with nucleases to prevent host immune reaction, as can be observed in tissue-derived ECMs (Crapo et al., 2011).

Decellularized CD-ECMs can then be used in their original format, fragmented (Carvalho et al., 2019b), grinded (Wei et al., 2015) or solubilized (Decaris et al., 2012). These formats give rise to 2D ECM layers or more complex 3D structures comprising 3D scaffolds (McAllister et al., 2009), spheroids (Cheng et al., 2009), fibers (Roberts et al., 2017), and sheets (Sharma et al., 2020; Figure 1B).

## Applications of CD-ECM

**FIGURE 2:**
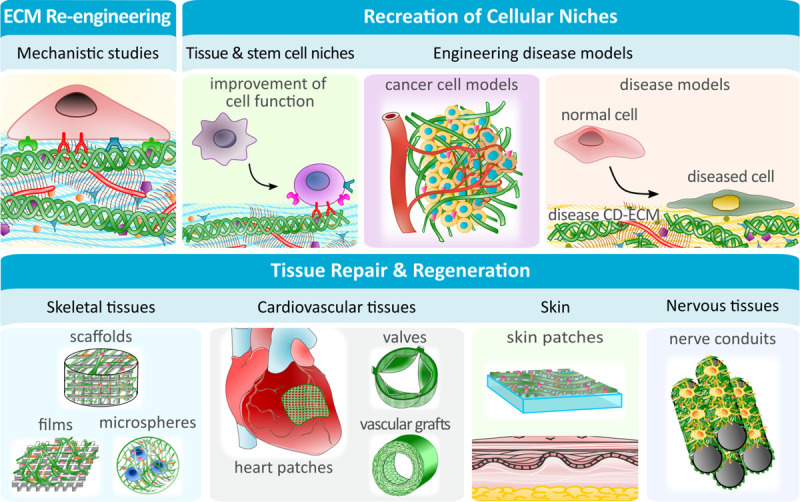
CD-ECM applications in fundamental research, pathophysiological studies and renegerative medicine. The ease with which CD-ECM can be modified, makes it the ideal platform to study detailed ECM mechanisms or the role of cellular niches under physiological and pathophysiological conditions. Specialized engineered cellular niches can be further utilized to improve cellular functions *in vitro*, such as stemness. In TERM, CD-ECMs can be created with specific mechanical and biological properties to be used on their own (i.e., as vascular grafts) or to enhance the performance of (semi-) synthetic biomaterials.

Numerous applications have been explored for CD-ECMs including the improvement of cellular functions, seen in tailored cellular niches, the study of ECM in a physiological and pathophysiological context, and the application in tissue engineering and regenerative medicine (TERM) (Figure 2).

### Recreation of Cellular Niches

#### Stem Cell Niches

The emulation of the native cellular microenvironment in culture is a prerequisite to maintain the cells’ phenotype and function. This is especially true for sensitive cell types, such as stem cells, which are known to undergo senescence and lose their stemness *ex vivo* (Hoshiba et al., 2016).

Various studies demonstrated that MSC-derived ECM can recapitulate the stem cell niche sufficiently to protect reseeded MSCs from oxidative stress, promote their proliferation, and conserve their stemness (Chen et al., 2007; Lai et al., 2010; Liu et al., 2016; Xing et al., 2020). CD-ECMs were also shown to maintain the native phenotype of neural progenitor cells (Yang et al., 2017; Hoshiba et al., 2018), embryonic stem cells (ESCs) (Klimanskaya et al., 2005), periodontal ligament stem cells (Xiong et al., 2019) and hematopoietic stem cells (Prewitz et al., 2013). Furthermore, ECMs derived from younger MSCs were shown to rejuvenate *in vitro-*aged and chronologically-aged MSCs (Pei et al., 2011; Sun et al., 2011; Lin et al., 2012). These effects were tightly linked to the biological profile of the ECM (reviewed in Sart et al., 2020).

#### Tissue-Specific Niches

Similar to MSC-derived ECM supporting stemness, ECMs derived from adipogenically or osteogenically induced MSCs promoted the respective lineage commitment of reseeded MSCs via integrated structural and regulatory proteins (Ang et al., 2014; Jeon et al., 2018; Carvalho et al., 2019a). Chondrogenic differentiation was best supported by chondrogenic ECM deposited in 3D (Cheng et al., 2009; Lu et al., 2011). Synovial MSC-derived ECM also protected chondrocytes from pro-inflammatory stimuli (Yan et al., 2020).

CD-ECMs from stromal, endothelial and epithelial cells could improve the function of specialized cell types, such as podocytes (Satyam et al., 2020), chondrocytes (Wei et al., 2015; Yang et al., 2018; Zhang et al., 2019), hepatocytes (Grant et al., 2017; Guo et al., 2017), Schwann cells (Xiao et al., 2016), as well as promote natural killer cell differentiation (Lee et al., 2020).

Similarly to adult stem cells, CD-ECMs synthesized by differentiating ESCs were able to promote early differentiation of ESCs, even without external factors (Goh et al., 2013). ECM produced by an endoderm-inducing cell line and ECM from liver progenitor cells promoted differentiation of pluripotent cells into insulin-expressing pancreatic β-cells (Higuchi et al., 2010) and hepatic cells (Kanninen et al., 2016), respectively.

Hence, CD-ECMs can be utilized to tailor cell and tissue-specific niches to promote cellular functions and study cell-niche interactions in detail.

### Engineering ECM in Disease

The ECM has a long-implicated role in disease development and progression, although the exact mechanisms often remain elusive. While the CD-ECM platform provides the opportunity to manipulate ECM and study it in detail, few studies utilized CD-ECM to study ECM mechanisms in disease (Raghunathan et al., 2018), most of them related to cancer.

It is currently well accepted that the tumor microenvironment plays a pivotal role in cancer cell behavior, including proliferation, invasiveness, metastasis and drug resistance (Serebriiskii et al., 2008). CD-ECMs provide the prospect to improve cancer models by recreating the cancer microenvironment using standard 2D, 3D cultures or more complex, organ-on-a-chip strategies (Gioiella et al., 2016; Kaukonen et al., 2017; Hoshiba, 2018). Indeed, culture of cancer cells on tumor CD-ECMs led to more physiologically relevant cancer cell phenotypes, as observed in various carcinoma (Serebriiskii et al., 2008; Eberle et al., 2011; Kaukonen et al., 2017), breast (Castelló-Cros et al., 2009; Hoshiba and Tanaka, 2013), and colon (Hoshiba, 2018) cancer models. Increased malignancy and drug resistance of cells was observed on invasive cancer CD-ECMs, in comparison to non-invasive cancer CD-ECMs (Hoshiba and Tanaka, 2013; Hoshiba, 2018). In contrast, upon culture on MSC-derived ECM, cancer cells proliferated less (Marinkovic et al., 2016) and showed reduced tumorgenecity upon implantation (Sun et al., 2010). Differences in cancer cell behavior were attributed not only to the biochemical composition of the tumor-associated ECM, but also to changes in stiffness (Kaukonen et al., 2016; Hoshiba, 2018) and a decreased cell adhesion (Hoshiba and Tanaka, 2013).

### Engineering and Characterization of CD-ECM to Study ECM Physiology

The ease of manipulating CD-ECM *in vitro* provides the opportunity to examine the reciprocal relationship between cells and their ECM.

Biochemical ECM re-engineering could be achieved through direct addition of functional groups (Xing et al., 2015) or exogenous factors (Sart et al., 2020), genetic modification (Higuchi et al., 2010) or growth factor stimulation of ECM-synthesizing cells (Wolchok and Tresco, 2012). Other changes in culture conditions, such as hypoxic cultures, were also shown to affect ECM properties and bioactivity (Hielscher, 2013).

Mechano-physical re-engineering could be achieved by culturing ECM-secreting cells in 3D sacrificial hydrogels (Yuan et al., 2018), on micro-molds (Schell et al., 2016), and micro- and nano-grooves (Ozguldez et al., 2018; Almici et al., 2020; Yang et al., 2020), forcing cell reorganization and leading to ECM assemblies with unique architectures (i.e., parallel fiber alignment). ECM postprocessing, such as cross-linking, could further alter ECM stiffness (Subbiah et al., 2016) or the overall presentation of CD-ECM. In particular, cross-linking of pepsin-solubilized CD-ECM with genipin resulted in the formation of hydrogels (Nyambat et al., 2020), Changes in biochemical and mechano-physical properties of the ECM let to changes in gene expression and behavior of reseeded cells (Kim et al., 2015; Ozguldez et al., 2018; Sart et al., 2020).

CD-ECM characterization and correlation with specific bioactivities can contribute to the mechanistic understanding of the ECM. ECM ultrastructure can be generally studied by scanning electron microscopy or atomic force microscopy (Kaukonen et al., 2016; Raghunathan et al., 2018). The latter method can also be used for biomechanical characterization (Prewitz et al., 2013; Assunção et al., 2020). Identification of proteins of interest is best performed by antibody-based assays such as immunocytochemistry or western blotting (Sart et al., 2020). Proteomic analysis based on mass spectroscopy enables the simultaneous identification of many components, however also faces challenges based on the insolubility and high complexity of the ECM (Ragelle et al., 2017; Senthebane et al., 2018; Silva et al., 2019). Furthermore, additional methods, such as Raman microscopy, can be used for biochemical characterization (Brauchle and Schenke-Layland, 2013).

### CD-ECM Applications in TERM

CD-ECM uses for TERM have been increasingly explored, either with CD-ECM alone or integrated in biomaterials. 3D scaffolds purely composed of CD-ECM were produced by decellularizing stacked cell sheets (McAllister et al., 2009) and pellets (Zwolinski et al., 2011), or depositing ECM in sacrificial materials, such as hollow tubes (ECM fibers) (Roberts et al., 2017) and foams (ECM porous scaffolds) (Wolchok and Tresco, 2010; Figure 1B).

For applications that require specific mechanical properties of the biomaterials, CD-ECM was integrated with synthetic materials, forming hybrid scaffolds (Schenke-Layland et al., 2009; Carvalho et al., 2019b; Sart et al., 2020). Hybrid materials met mechanical requirements, while providing adequate biochemical stimuli, thus facilitating implant integration and functionality (Silva et al., 2020). Commonly, CD-ECM was utilized as a coating by simply decellularizing cells on the biomaterial surface (Kumar et al., 2016; Junka et al., 2020), although solubilized CD-ECM was also used as a coating (Decaris et al., 2012). A more sophisticated approach introduced azide-modified monosaccharides into culture media, which subsequently were incorporated into the ECM. The CD-ECM could then be covalently “clicked” to material surfaces (Ruff et al., 2017). Alternative approaches directly incorporated CD-ECM into the biomaterial during synthesis (e.g., electro-spinning) (Schenke-Layland et al., 2009; Carvalho et al., 2019b).

CD-ECMs based biomaterials were mainly investigated for skeletal and cardiovascular repair, although other applications such as in skin (Suhaeri et al., 2018) and peripheral nerve repair (Gu et al., 2017) were also explored.

#### CD-ECM for Skeletal Repair

Most approaches to engineer CD-ECM-carrying bone implants utilized inorganic materials (reviewed in Zhang et al., 2016), such as meshes and scaffolds (Kang et al., 2012; Antebi et al., 2015; Kim et al., 2015; Jeon et al., 2016; Kumar et al., 2016; Noh et al., 2016; Junka et al., 2020; Silva et al., 2020). These were coated with ECM assembled by collagen I-overexpressing epithelial cells (Noh et al., 2016), fibroblasts (Kim et al., 2015), MSCs (Kang et al., 2011; Silva et al., 2020), endothelial cells (Kang et al., 2012), osteoblasts (Jeon et al., 2016; Kumar et al., 2016) and combinations thereof (Junka et al., 2020). CD-ECM coated scaffolds promoted attachment, proliferation, and bone-like tissue formation *in vitro*. In a more advanced approach, Kim et al. (2015) enhanced an PLGA/PLA-based mesh scaffold coated with CD-ECM, by covalently conjugating heparin to the ECM. The heparin then acted as a growth factor reservoir for bone morphogenic protein-2 (BMP2), thereby promoting bone healing *in vivo* (Kim et al., 2015). CD-ECM was also used to increase retention of osteogenically precommitted MSCs on biomaterial surfaces after implantation. This revitalized ECM successfully repaired mouse calvaria defects (Zeitouni et al., 2012).

Therapeutic approaches targeting cartilage repair mainly utilized 3D scaffolds purely composed of CD-ECM (Jin et al., 2007; Tang et al., 2013, 2014) or CD-ECM-loaded hydrogels (Yuan et al., 2013). Indeed, 3D scaffolds of chondrocyte- and MSC-derived ECM reseeded with chondrocytes induced ectopic hyaline-like cartilage formation *in vivo* (Jin et al., 2007; Tang et al., 2013). When applied to an osteochondral defect together with bone marrow stimulation, autologous MSC-derived ECM could enhance cartilage repair (Tang et al., 2014). In another study, a protective effect on the degenerating cartilage could be demonstrated, when collagen I microspheres containing nucleus pulposus CD-ECM and MSCs were injected into a rabbit degenerative disc model (Yuan et al., 2013).

#### CD-ECM for Cardiovascular Tissue Engineering and Repair

CD-ECMs have been explored as cardiac patches for cell-delivery (Schmuck et al., 2014; Kim et al., 2019), as well as to engineer heart valves replacements (Weber et al., 2013) and blood vessel grafts (McAllister et al., 2009; Syedain et al., 2014; Xing et al., 2017).

Cardiac patches were composed of fibroblast ECM alone (Schmuck et al., 2014) or combined with a polyvinyl alcohol sheet, resulting in a stretchable scaffold for cell delivery. Application of the latter in a rat myocardial infarct model resulted in improved cardiac remodeling (Kim et al., 2019).

A cardiac valve prototype containing vein-derived fibroblast ECM was implanted in a non-human primate. Albeit valve functionality was reduced, there was a significant improvement in repopulation by host cells, when compared to decellularized human heart valves (Weber et al., 2013).

McAllister et al. (2009) utilized partially devitalized autologous fibroblast/endothelial CD-ECM sheets to form vascular access grafts for dialysis patients. Complete remodeling and repopulation of CD-ECM occurred, although diffuse dilation of the graft was observed (McAllister et al., 2009).

In order to improve this low graft resistance, Syedain et al. (2014) stimulated tubular fibroblast cultures in a pulsed-flow-stretch bioreactor. Upon implantation of the decellularized graft into the femoral artery of sheep, no dilation was observed. Once completely recellularized, the grafts resembled native vessels in terms of cellular composition, ECM architecture and mechanical properties (Syedain et al., 2014).

## Conclusion and Outlook

Although CD-ECM was continuously explored for over three decades and many safety concerns associated with tissue-derived products can be addressed, relatively slow advancements were made over the years. This can be partially attributed to the low amounts of CD-ECM that can be harvested *in vitro*, indicating that strategies for upscaling processes as well as manufacturing of larger 3D constructs need to be developed.

In addition, most TERM approaches used unmodified ECM from MSCs or tissue-specific cell types to induce cellular responses *in vitro* and *in vivo*. And although various approaches on how to re-engineer the CD-ECM are proposed, relatively few are applied to address scientific questions or to manufacture biomaterials with enhanced desired bioactivities. The reason for the limited progress can be partially attributed to our restricted fundamental understanding of the ECM. Hence, functional studies in combination with CD-ECM characterization will have to be adopted. Another reason is that re-engineering approaches are mainly focused on biological manipulation. Research at the interface to other disciplines such as materials science is indeed required to enable further evolvement of the CD-ECM research field. Future applications could focus on bio-inks with tailor-made bioactivities for 3D bioprinting or improved biomimetic cell niches in organ-on-a-chip approaches.

In conclusion, CD-ECM based research is far from its full potential. Advancements in methodologies as well as innovative interdisciplinary approaches are needed to pave the way for an exciting next generation of CD-ECMs for basic research and therapeutic approaches.

## Author Contributions

All authors contributed to the elaboration of this review.

## Conflict of Interest

The authors declare that the research was conducted in the absence of any commercial or financial relationships that could be construed as a potential conflict of interest.
